# Assessment of long-term neurodevelopmental outcome following trials of medicinal products in newborn infants

**DOI:** 10.1038/s41390-019-0526-1

**Published:** 2019-08-09

**Authors:** Neil Marlow, Lex W. Doyle, Peter Anderson, Samantha Johnson, Varsha Bhatt-Mehta, Giancarlo Natalucci, Brian A. Darlow, Jonathan M. Davis, Mark A. Turner, for the International Neonatal Consortium (INC)

**Affiliations:** 1 Neonatal Medicine, University College London, Elizabeth Garrett Anderson Institute for Women’s Health London, London, UK; 2 Department of Obstetrics and Gynecology, The Royal Women’s Hospital, University of Melbourne, Melbourne, VIC Australia; 3 Turner Institute for Brain and Mental Health & School of Psychological Sciences, Monash University, Clayton, VIC Australia; 4 Department of Health Sciences, University of Leicester, Leicester, UK; 5 C.S.Mott Children’s Hospital, University of Michigan, Ann Arbor, MI USA; 6 Department of Neonatology, University of Zurich and University Hospital Zurich; Child Development Center, University Children’s Hospital Zurich, Zurich, Switzerland; 7 Department of Paediatrics, University of Otago, Christchurch, New Zealand; 8 Department of Pediatrics, Floating Hospital for Children, Tufts Medical Center, Boston, MA and Tufts Clinical and Translational Science Institute, Tufts University, Boston, MA USA; 9 Institute of Translational Medicine, University of Liverpool, Liverpool, UK; 10 Critical Path Institute, Tucson, AZ USA

## Abstract

There is significant uncertainty over the role of assessment of long-term neurodevelopmental outcome (LTO) in neonatal clinical trials. A multidisciplinary working group was established to identify key issues in this area and to make recommendations about optimal approaches to evaluate LTO in therapeutic trials in newborns, which can be developed by sponsors and investigators with other key stakeholders. A key consideration for neonatal trials is the potential for the investigational product to cause widespread effects and drives the need to assess outcome in multiple organs. Thus investigators must assess whether the product has an impact on the brain and the potential for it to cause potential effects on LTO. Critically, is assessment of LTO an important direct therapeutic target or a safety outcome? Such decisions and outcomes need to be specific to the product being studied and use published data, only considering expert opinion when prior evidence does not exist. In designing the trial, the balance of benefits, costs, and burdens of assessments to the researcher and families need to be considered. Families and parent advocates should be involved in design and execution of the study. A framework is presented for use by all key stakeholders to determine the need, nature, and duration of LTO assessments in regulatory trials involving newborn infants.

## Introduction

The effect of an investigational medicinal product (IMP) or other interventions administered to newborn infants may be detected well beyond the neonatal period. The extent of the affected domains has been set out in a report of a workshop held in 2011.^1^ Recent advances in neuroprotection following intrapartum hypoxia at term have highlighted the complexity of long-term neurodevelopmental outcome (LTO) from hypoxic–ischemic encephalopathy (HIE).^2,3^

The study of most pharmacologic and non-pharmacologic interventions in the newborn infant will require monitoring of outcomes beyond discharge from inpatient neonatal services. This is needed to detect the primary outcome, to confirm persistence of neonatal effects of the intervention, or to demonstrate long-term safety.^4^ Rapid and continued development as well as the non-specificity of medicines used in the neonatal period means that investigational products rarely have precisely targeted effects. Products given in the neonatal period may subtly alter the developmental trajectory of organ development, for example, lung or vascular development,^5^ such that the effects are only detectable as the organ system matures. This phenomenon is compounded by the effects of factors other than the study intervention on development and maturation as well as a lack of reliable early biomarkers of effectiveness or adverse effects for most conditions that appear in children after neonatal illness.

This narrative review considers factors associated with neonatal LTOs while drawing attention to other domains when appropriate. It reflects the views of a broad spectrum of individuals from clinical practice, industry partners, and regulators.

## Neonatal long-term outcome studies

In general, long-term follow-up is often conducted in studies that are separate yet coordinated with the initial clinical trials. Long-term studies are not easy to do as they require tracking of children and their families after discharge from hospital for several years or more, which can be expensive and overly burdensome.^6^ Trials of investigational products in newborn infants may need to include suitable tracking and retention strategies as part of the licensing process, unless it can be linked to routine LTO follow-up that is part of the standard care. Dropouts in long-term outcome studies are common in both academic and industry-based studies—and they are rarely at random, preferentially affecting children with adverse outcomes^7,8^ and those from socially disadvantaged backgrounds.^9,10^ Trial size calculations for long-term studies (e.g., within post-approval registries) need to account for a realistic proportion of dropouts in order to ensure adequate data quality.

Certain effects may not be detectable for many years. For example, lung function tests are not reliable until later in childhood^11^; executive functions, the building blocks of cognitive function in childhood and adult life, differentiate into separable functions that are more reliably assessed at school age^12^; changes in vascularization affecting blood pressure may not reliably be detected until early adolescence.^13^ While important outcomes may not manifest until 5–10 years after exposure, it is not practical to wait this long for licensing/marketing authorization.

These considerations mean that it is important to determine which LTOs are central to licensing and which will inform post-marketing assessments. Direct evidence of clinical benefit is usually required for licensing, unless a validated surrogate endpoint can be identified. Many assessments currently conducted during the neonatal period are not specific or sensitive predictors of important clinical outcomes, either as benefit or harm, and so are unlikely to provide validated surrogate endpoints.

Delays and difficulties often arise during long-term outcome studies. If long-term studies are unnecessarily included in the initial assessment for licensing or marketing authorization, there may be delays in approval that prevent infants from accessing a useful product. Delays can also lead to reduced feasibility because off-label drug use removes equipoise and because delays can cause investigator and parent fatigue. However, longer-term assessments may be essential to establish both safety and efficacy of some investigational products.

The decision about which outcomes to use as the basis for neonatal licensing should be made early during the process to approve the pediatric development plan or its constituent studies. The role and nature of shorter-term outcomes will depend on the natural history of the condition, the known and expected properties of the product, and the specific neonatal population that is investigated. The licensed indication may not be the same as the clinical diagnosis. Outcomes need to be relevant to families and clinicians (and other stakeholders such as reimbursement agencies). The sponsor should present a well-reasoned proposal for which outcomes should contribute to licensing with a specific focus on outcomes that should be included in Pharmacovigilance Risk Management Plans or adaptive licensing. Short-term risks and benefits may not be concordant with long-term outcomes. For example, cranial ultrasound may not detect abnormalities in infants who subsequently develop cerebral palsy, neurocognitive, or behavioral problems. However, potential discordance is a justification for well-designed surveillance that makes account of this possibility, not for delaying licensing once evidence of a useful clinical effect is apparent. A transient benefit seen over 1–2 years that is not reflected in later life may still be valued by families and payers.

Understanding the multiple influences that are active during childhood is equally important, as these may confound the results of clinical trials. For example, males have worse outcomes than females in many neonatal studies. Family structure may influence how a child acquires skills, as may the language spoken at home (if more than one), particularly when testing is done as structured language is appearing in the second and third years of life. Finally, the socio-economic status of the family and availability of interventional services may modify both short- and long-term outcomes. Data collection should capture these influences as it is necessary to include sensitivity analyses as secondary exploratory analyses. In large multicenter, randomized, controlled trials, sample size and randomization should be robust enough to ensure even allocation of confounders between the study groups. However, randomization does not necessarily influence the balance of confounders that may arise subsequently (e.g., meningitis occurring in infancy or new onset of seizure disorders; both may affect neurodevelopment).

## Framework for planning LTO evaluations

This section presents general points to be considered when assessing the need for, and structure of, LTO evaluations for neonatal trials. Points to consider may include:

1. *Justify all trial design components according to the specific trial drug*—This will include the investigational product itself, the indication for use of the product, and the population where it will be tested. Other interventions, such as the use of therapeutic hypothermia during HIE, should be reflected in the justification. All components should be justified using data, reverting to expert professional opinion only when necessary.
2. *Account for the nature of the outcome as a primary target or safety consideration*—where the outcome is a direct consequence of the intervention, a formal evaluation of a defined LTO is necessary, tailored to detect the proposed effect (Fig. 1a). Where the investigational product may be associated with adverse LTO, this may not necessarily be a direct consequence of the treatment effect but other causal pathways. In this instance, it is safer to evaluate all outcome domains (Fig. 1b). There is a co-dependency of adverse outcome domains primarily because many causal pathways are common. The pathogenesis of poor neurodevelopmental outcomes is complex and many prenatal and postnatal influences combine with the child’s environment to produce a particular outcome. These influences will usually be evenly segregated between trial groups through the randomization process. In some trials, it may be appropriate to ensure even recruitment through stratification or minimization techniques to account for the confounding influences that are identifiable at study entry. Examples of important confounders for LTO are male sex, gestational age, fetal growth, neonatal brain injury, and socio-economic status.^14–16^ Trial data should include all of these potential confounders.

**Fig. 1:**
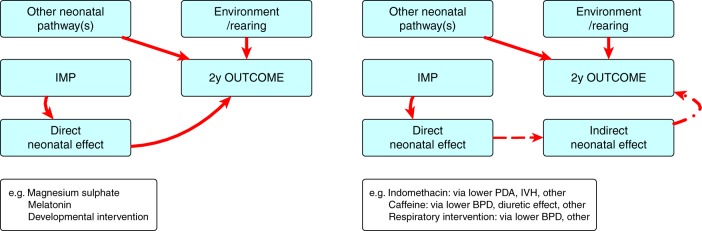
Causal pathways to adverse long-term outcomes and needs for assessment. **a** Where the outcome is a direct consequence of the intervention, a formal evaluation of a defined long-term neurodevelopmental outcome (LTO) is necessary, tailored to detect the proposed effect. **b** Where the outcome is an indirect consequence of the intervention, it is safer to evaluate all domains of relevant LTOs
3. *Balance the benefits, costs, and burden of the assessments*—these may be balanced against the assessments themselves, the intervention, the indication, and the population (including co-morbidities).
  With respect to each time that contact is made with a family, it is important to consider:
  - The burden on the participant and family
  - The burden on the health-care system both during the trial and subsequently if the role of the intervention changes after the trial (i.e., use becomes more generalized)
  - Costs to the family (direct and indirect) against utility for the sponsor. With respect to the intervention, consider:
  - Benefits against risk
    i. Anticipated
    ii. Potentially unanticipated
    iii. The magnitude of the benefit or risk
    With respect to the population, consider:
  - What are the inherent risks in the population (the impact of the initial disease process on LTO) that the investigational product seeks to mitigate?
  The direct costs and resources required for long-term follow-up should be identified and guaranteed before the trial starts. Sponsors, clinicians, academics, and relevant advocacy groups should all facilitate long-term follow-up by designing the studies carefully and by contributing to/advertising the studies when appropriate. Health-care systems should provide support to long-term follow-up studies by providing suitable infrastructure that promotes long-term follow-up—which in any case is essential for the responsible clinical care of newborn infants (especially preterm infants) and by maintaining systems that provide unique identifiers. The collection of high-quality data in routine clinical practice may also contribute to LTO assessments but requires standardization and coordination across multiple settings that may not be feasible.
4. *Involve families and parent advocates in the study design*—this should be proportional to the design and involvement of the trial and likely benefit. As a general principle, the input of families will assist in ensuring that the trial is relevant, acceptable, and feasible to service users. It is acknowledged that sometimes such input may lead to alteration of commonly used assessments: e.g., specific parental questions or instruments. For example, parents may request that certain questions be removed from standard questionnaires because they appear to be insensitive in current usage, which may change the overall psychometric properties of the instrument. However, the input of families is often of great benefit in ensuring that the risk/benefits/burden of the trial is optimized. Where such input is sought, resources need to be made available to participants to facilitate their input, including reimbursement for their time and travel.
5. *Account for the nature of the intervention*—in terms of likely effects and use:

- What is known about drug disposition in the target group (absorption, distribution, metabolism, excretion, toxicity, integrity of the blood–brain barrier)?
- What is known of toxicology in appropriate preclinical models?
- Are there likely effects from excipients or formulation strategies?
- How is the drug to be administered?
- What is known about the neurological impact in terms of benefits or harms, the likelihood of these impacts, and the extent to which these can be excluded as issues?

6. *Account for the background morbidity in the population*—medicines rarely remove all morbidity in clinical populations as most morbidities have complex and multifactorial etiologies. In any population, there will be background morbidities which may confound the effects that are being studied. Thus, particularly when attempting to evaluate the effects of drugs on LTO, the population baseline outcomes must be known and will differ—some examples of populations with very differing risks may be:
  - Term infants with suspected infection
  - Term infants recruited to vaccine studies
  - Term infants with HIE
  - Preterm infants ≤32 weeks postmenstrual age with an oxygen requirement
  - Preterm infants ≤26 weeks with poor perfusion and/or hypotension
7. *Account for the nature of the trial*—several types of investigation are relevant to these considerations, including regulated submission for marketing authorization/labeling, contribution to prescribing (strategic trials), and to health technology assessment. Data from trials may be relevant to other trial situations and data should be re-used for other relevant purposes if at all possible, to minimize the “ask” of families. Furthermore, the studies and data required to obtain marketing authorization may not be suitable to change clinical practice or justify reimbursement, and the sponsor may feel it is necessary to add assessments beyond these minimal requirements. However, each additional assessment must be thoroughly justified.
8. *The plan for LTO evaluation*—Following consideration of the above issues, a bespoke plan can be made or one or more of the following plans may be followed:
  - No specific long-term follow-up is necessary beyond the initial hospital discharge
  - Studies should retain a unique identifier to allow record linkage with routine health data
  - Studies should retain contact with the family and ask general questions about their health status
  - Studies should screen participants for important LTO: assessments should be established a priori and any positive screens evaluated if/when they occur
  - There should be formal follow-up of all participants at appropriate time intervals to directly evaluate their clinical and neurodevelopmental status
  - Follow-up should be conducted using a registry (new or existing) that includes participants in studies recruiting during pregnancy or the neonatal period. This could include other infants exposed to the IMP.

The plan will be different depending on the feasibility of ongoing surveillance, the relative prevalence of anticipated outcomes in different populations, and on the likelihood of brain or other organs having adverse outcomes.

## Technical issues in planning evaluation of LTOs

The online Supplemental Material describes the considerations and details of specific neurodevelopmental assessments, drawn from professional consensus and previous studies, with explanatory notes on the reasons for choice of measure, and categorization of outcomes for safety purposes. These categories are used worldwide as outcomes for a range of clinical and medicine-related studies and are especially relevant in neonatal trials. If an investigational product is to achieve clinical acceptability, the quality and nature of the outcome assessment should be understood by the entire clinical community with reference to common practice. That is not to say that innovative outcomes are not of value, but that they must be set in context and be of sufficient validity, reliability, and quality to have confidence in clinical applicability. Further, specific products may need tailored outcomes depending on the target and the nature of the organ effect that is being studied.

With respect to neurodevelopmental outcomes, trials may be grouped as those where the investigational product is targeting specific neurological outcomes or where general safety monitoring is required, which could include neurodevelopmental outcome. Although specific recommendations of assessment methods are made (in an attempt to standardize and harmonize approaches), these examples are not intended to preclude the use of other instruments or assessment tools.

## Ages at assessment

**Fig. 2:**
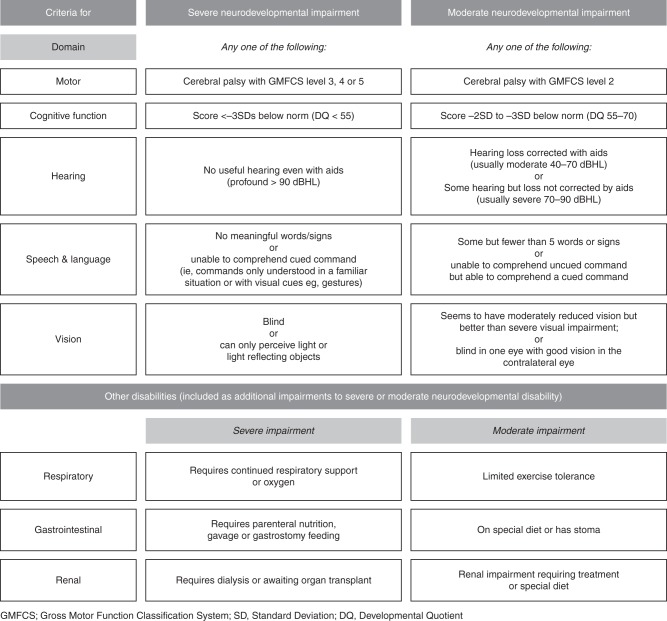
Example of a consensus scheme for categorization of health status at 2 years of age, including neurodevelopmental outcomes^17^

Because of the lack of robust biomarkers for LTOs, which are needed, it is necessary to evaluate trial participants up to an age at which the evaluation identifies reliable indicators of long-term outcomes. For efficacy of neurologically focused interventions or for assessment of neurological safety, the primary outcome should be evaluated at 2 years of age (corrected for prematurity if appropriate), as the focus of the first phase of LTO monitoring. Such an assessment is performed by many neonatal teams for audit and quality-control purposes in high-risk cohorts and may be adapted during data collection for trials. The rationale and recommended classifications have been published (Fig. 2) and the assessments are discussed in greater detail in the web supplement. These should be supplemented with treatment-specific outcomes as appropriate (e.g., seizure frequency in the study of an anti-epileptic medication). These outcomes may be considered necessary to support licensing where the effects of the investigational product are not directly quantifiable during the neonatal period.

To rely completely on 2-year (or even earlier) outcomes runs the risk of misclassification of final outcome and of missing more subtle cognitive, behavioral, or sensorimotor outcomes. Assessments performed at 2 years corrected gestational age are performed when many pathological outcomes are still differentiating as part of the normal child development (Fig. 2). Accurate ascertainment of neurocognitive, academic, and behavioral assessments requires children to be followed until later in childhood when school performance can be better assessed. All of these conditions are more accurately measured at later ages and tend to differentiate during the first 4–6 years after birth. From early school age, cognitive scores tend to track in individuals and more precise definition of motor and sensory outcomes is possible. Where appropriate, these assessments should be combined with treatment-specific measures to enhance the understanding of the very long-term effects of the product. While outcomes at 5–6 years of age are important confirmatory assessments, generally they should supplement licensing processes and are not necessarily required for initial licensing because of the lag time to their detection.

In some settings, it may be prudent to collect routine data from national systems to support safety and very long-term outcomes—examples of this may be ongoing mortality or cancer registration monitoring or the results of standardized national educational attainment tests. As most studies are carried out in international settings, care needs to be taken when proposing to use such assessments as these may not be an adequate substitute for research-specific assessments and highlights difficulties in comparing outcomes between different health-care and education systems. This may be due to inconsistencies in granularity or data quality or in the scope of geographic coverage relative to trial recruitment. Nevertheless, these are potentially important surveillance tools and may identify important long-term risks when appropriate. In addition to these assessments, it may be prudent to add additional focused assessment to support licensing of particular products. All ongoing data collection should meet relevant national data protection regulations, which should be factored into trial planning and delivery.

## Post-discharge deaths

At each assessment point, the number of patients who have died and the principal reason for death should be recorded, together with any pre-existing identified disabilities. Deaths may confound some trials and any assessment plans should indicate how deaths are to be treated in the analysis, even if the product is not thought to directly relate to the death. Since death is often a competing outcome, it is not uncommon to see combined primary outcome measures, which include death or another serious morbidity.

## Conclusion

**Fig. 3:**
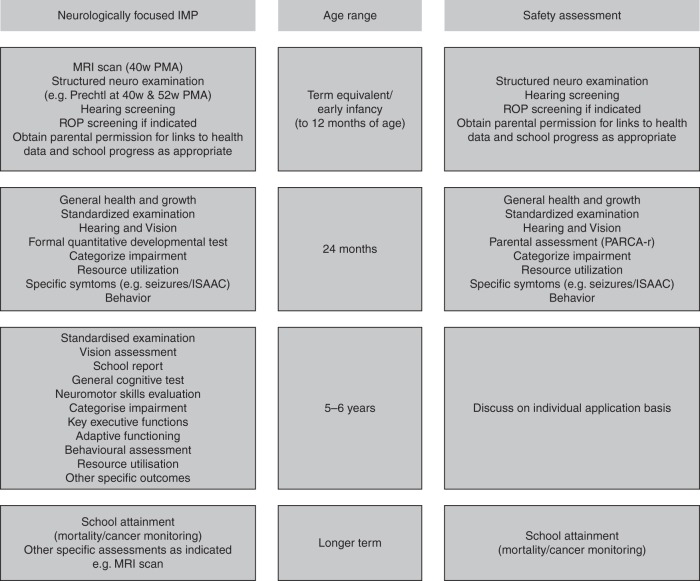
Summary of proposals for long-term neurodevelopmental outcome assessment strategy

Clinical trials of IMPs in the neonatal period may require long-term evaluations for assessment of both therapeutic and safety signals. Drugs may have negative effects on the infant, both directly and unanticipated, which may be detected early in the neonatal period or later at school age. This paper conceptualizes recommendations for the examination of long-term outcomes and proposes a framework within which more specific outcomes pertinent to the investigational product may be added (Fig. 3).

## Supplementary information

Supplementary Information
