# Supplementary material for: Assessment of long-term neurodevelopmental outcome following trials of medicinal products in newborn infants
Source: Pediatr Res. 2019 Aug 9;86(5):567–72. doi: 10.1038/s41390-019-0526-1 (PMC6848023; doi:10.1038/s41390-019-0526-1)
Supplement: Supplementary file 1 — Supplementary Information [file 41390_2019_526_MOESM1_ESM.docx]

**On-Line Supplemental Material**

**For:**

**Assessment of Long Term Neurodevelopmental Outcome Following Trials of Medicinal Products in Neonates**

Marlow, N^1^, Doyle, LW^2^, Anderson, P^3^, Johnson, S^4^, Bhatt-Mehta, V^5^, Natalucci, G^6^, Darlow, BA^7^, Davis, JM^8^, and Turner, MA^9^ for the International Neonatal Consortium (INC)

^1^Neil Marlow DM, Professor of Neonatal Medicine, UCL, London, UK

^2^Lex W Doyle, MD, Department of Obstetrics and Gynaecology, The Royal Women’s Hospital, University of Melbourne, Australia

^3^Peter Anderson, PhD, University of Melbourne, Australia

^4^Samantha Johnson, PhD, Department of Health Sciences, University of Leicester, UK

^5^Varsha Bhatt-Mehta, Pharm.D., MS, FCCP, College of Pharmacy and Michigan Medicine, University of Michigan, US

^6^Giancarlo Natalucci, MD, Department of Neonatology, University of Zurich and University Hospital Zurich; Child Development Center, University Children’s Hospital Zurich, Switzerland

^7^Brian A. Darlow, MD, University of Otago, Christchurch, New Zealand

^8^Jonathan M. Davis, MD, Department of Pediatrics, Floating Hospital for Children, Tufts Medical Center, Boston, MA and Tufts Clinical and Translational Science Institute, Tufts University, Boston, MA

^9^Mark A. Turner, PhD, MRCPCH, Institute of Translational Medicine, University of Liverpool, Liverpool, UK

**Address for correspondence:**

Neil Marlow DM FMedSci

Professor of Neonatal Medicine
UCL EGA Institute for Women's Health
74 Huntley Street
London WC1E 6AU
United Kingdom

Email:  [n.marlow@ucl.ac.uk](applewebdata://156231EB-E224-4DBF-ADD5-794CF43F1C77/n.marlow@ucl.ac.uk)

Tel: +44 (0) 20 7679 0834 (PA: Carla Logon)
Fax: +44 (0) 20 3108 2036

**The following sections provide important details of the timing and content of follow up assessments.**

Formal assessment is carried out at:

- Discharge from hospital (and/or at standardized PMA timepoints)
- Early infancy
- Two years of age corrected for prematurity
- Later assessments

1. **Discharge from hospital, and/or at standardised PMA timepoints** – in addition to perinatal and neonatal demographics, a baseline assessment should be performed so that any bias in the proportion lost to follow up can be ascertained based on a fixed point of discharge from the neonatal service. This should comprise a baseline assessment using:
   - Neurology:
     - Head circumference
     - Presence of seizures
     - Standardized neurological examination, e.g. the Amiel Tison or the Hammersmith Infant Neurological Examination or the General Movements Assessment (see below)
     - Brain MRI scan: it is unclear whether routine MRI scanning at term equivalence will provide more prognostic information compared with a cranial ultrasound.^1^ Certain pathologies, for example cerebellar hemorrhage, are challenging to detect with ultrasound and may have important implications for later risk of neurocognitive outcomes.^2^ Summary scores for MRI and ultrasound provide baseline data on which potential bias through dropout during extended follow up may be evaluated.
     - Feeding – record whether supplemental gavage feeds or intravenous nutrition are required
   - Respiratory – record age last receiving supplemental oxygen and whether the infant was discharged home with supplemental oxygen or any form of assisted ventilation
   - Growth – expressed as standard deviation score for length and weight using consistent standards across sites (bearing in mind that “intra-trial” comparisons between allocations are important)
   - Results of inpatient hearing and vision screening (as appropriate)
   - Medications
   - Permission should be sought to: a) contact the parents (and other family members or care givers) about subsequent follow-up assessment, and b) access to routinely collected health and education data (if these are collected and available across a majority of sites)
   - **Sociodemographic data** should be defined and prospectively collected at each assessment to confirm any changes in status. Particular interest should be given to non-biological factors that are known to affect LTOs of interest.
2. **Early Infancy**
   - Record results of hearing and vision screening tests
   - Consider using assessment of MRI findings, General Movements – video assessment evaluating the presence of ‘fidgety movements’ at 3-4 months corrected gestational age – and other assessments as a predictor of cerebral palsy^3^, bearing in mind the limitations of these techniques and that cerebral palsy is only one important outcome.
   - Consider the use of symptom diaries/parental questionnaires for respiratory symptoms, medications, seizures, etc
3. **2 years corrected for gestational age (CGA)**

There are several schemes for categorizing outcome at two years CGA. The following domains should be evaluated in all studies that make a formal assessment:

- **Neuromotor:** Each child should have a formal neurological examination and assessment of fine and gross motor function. Children who have a neurological abnormality should be classified according to a standard grid, such as that proposed by the Surveillance of CP in Europe group (SCPE; [www.scpenetwork.eu](http://www.scpenetwork.eu)). Their simple approach provides consistent classification into spastic (bilateral, unilateral), dyskinetic (dystonic, choreo-athetoid), ataxic, or other forms of CP. Functional outcome in children with CP is increasingly important. Simple classifications that describe functional outcomes, such as the Gross Motor Function Classification System (GMFCS)^4^ and the Manual Abilities Classification System (MACS)^5^ are invaluable approaches to maintain consistency between reports, patients, and clinical trial data and should be ascertained.
- **Developmental assessment:** Each child should have a formal assessment of developmental status.
  - - For studies with neurological targets, this should be carried out using a standardized and validated face-to-face assessment, such as the Bayley Scales of Infant and Toddler Development 3^rd^ Edition^6^ (Bayley-III) or the Griffiths III^7 8^, or equivalent. These developmental tests produce standardized summary scores for multiple domains of the child’s development. Scores can be compared between study groups to evaluate the effect on developmental progress. Such tests have reasonable predictive value for later general cognitive scores^9 10^, but are not formal Intelligence Quotient (IQ) tests. Formal developmental tests such as these require training and validation to ensure the details of the assessment proforma are adhered to; continual re-validation of assessors should be part of the study protocol.
    - Generally, these tests should be formally adapted or translated for the local population, preferably by the test developer, and validated with respect to the construct of interest (e.g. general cognitive function or IQ, sustained attention, etc). The tests should have quantified acceptable psychometric properties, including reliability, and have local standardization within the previous 10-15 years. Where no such locally validated tests exist, a normative sample should be tested alongside the study group to provide reference values. Comparisons between study arms are important. The use of standardized mean differences may allow results from more than one test to contribute to comparisons between study allocations.
- Where standardized language tests are applied to infants of non-native speaking families as part of general developmental tests, robust translations should be sought, or consideration given to using non-verbal tests to determine cognitive outcome.
  - - For studies in which there is safety monitoring of outcomes (e.g. trials of respiratory medications), formal developmental testing may be carried out. However, the use of well-validated parental reporting scales has definite advantages in terms of cost and time. There are several appropriate developmental screening assessments such as the Ages and Stages Questionnaire (ASQ-3) or the PARCA-R (Parent Report of Children’s Abilities – revised; www.parca-r.info), or similar^11^.
- **Hearing and vision:** results of hearing and vision testing should be recorded together with a functional history for both systems (see classification below). Use of aids (hearing, glasses) should also be reported
- **Communication/language:** Language skills are quantified as part of developmental testing. A classification for children with moderate or severe impairment of communication skills is provided below (taking into account various ethnic differences and whether one or more languages are spoken in the home).
- **Behavior:** Behavior is difficult to assess at two years as the common behavioral syndromes have not differentiated at this stage and most assessments are therefore difficult to interpret. There is a range of standardized tools that may be used (e.g. Child Behavior Checklist (CBCL 1.5-5)^11 12^, Infant Toddler Social Emotional Assessment and its Brief form (ITSEA^13^, BITSEA^14^), Modified Checklist for Autism in Toddlers (M-CHAT; https://m-chat.org), or Strengths and Difficulties Questionnaire^15^ (SDQ 2-4)). Many follow up programs also screen for autism-like symptoms or other behavioral problems in preterm populations, for whom the prevalence is increased.^16-18^ If such screening is undertaken then follow up examinations are required for children who screen positive. The proportion who are assigned a diagnosis after a formal assessment for autism or other behavioral problems should then be reported, as opposed to just the proportion who screen positive. These children should be referred for relevant support services (e.g. early intervention). The implementation of the trial should account for the need to assess children who screen positive, given that many such children will not be under routine follow-up by the relevant services
- **Resource utilization evaluation** needs to be included if the data will be used to support economic analysis, for example by health technology assessment agencies, payers etc.
- **Other organ-specific outcomes** (e.g. respiratory symptoms and therapy)
- **General health and growth**

1. **Categorisation of neurodevelopmental outcomes at two years**

Formal categorization of outcomes varies slightly between different health systems. We prefer to categorize 2 year old children as having severe, moderate or no/mild impairments, as opposed to a single impaired category. The consensus scheme that has been adopted in the UK is shown in Figure 3.^19 20^ This or a similar scheme could be used to categorize outcomes. Note that for some developmental tests (e.g. Bayley-III) different cut offs are recommended.^21^ Such categories have been widely used in North America, Europe and Australia. These categories may be inconsistent, leading to apparent similarity between very different impairments producing different forms of disability. For this reason, each trial should define a single, comparable outcome as the focus of reporting for each assessment. For some studies, elements may be selected as primary outcomes, e.g. cerebral palsy in trials of neuroprotection^22^, but all components should be reported.

When these categories are combined with death in a composite outcome (“death or disability” or “survival without neurodevelopmental impairment’), death may confound the outcomes. Care should be taken as occasionally the two dimensions of death and disability may act in different directions. Careful statistical analysis and transparent reporting of the rates of individual components of any composite are critical to understanding the outcomes described.

It should be noted that non-neurological disability may be an equally important outcome and some suggested definitions are also included in Figure 3. These may need to be adapted in different trials but should conform to the International Classification of Functioning Disability and Health (ICF; [www.who.int/classifications/icf/en/)](http://www.who.int/classifications/icf/en/)). They should also be consistent with the adult-focused WHO Disability Assessment Schedule (WHODAS 2.0; [www.who.int/classifications/icf/more_whodas/en/](http://www.who.int/classifications/icf/more_whodas/en/)) for the assessment of older children (see below).

Some children will not be able to complete all neurological or other assessments due to behavioral issues or significant physical or cognitive impairment. These situations can be anticipated in this population and procedures for assigning a score or classification to these children should be agreed prospectively and communicated to the assessors.

1. **Assessment at later ages**

Although 2-year outcomes may be considered sufficient to support licensing, 2-year outcomes may not precisely predict outcomes at later ages.^23^ Furthermore, correlation between cognitive scores is much higher from school age through to adult life^9 24^ and considerably better than prediction from developmental scores at 2 years CGA. Therefore, it seems prudent to re-evaluate children at a later age, when categorization is likely to be more accurate and increased detail on cognitive performance and behavioral outcomes is available. This will comprise the largest group of adverse outcomes for preterm and other clinical populations of neonates.  It has the advantage of confirming any effects seen at two years and evaluating any unanticipated or new findings. The disadvantages of this approach are that there is likely to be increased dropout, the cost of carrying out such evaluations is high, and intercurrent confounding variables (e.g. environmental factors) may alter the size of the effect. Generally, children should have been in school for at least 12 months prior to the assessment (particularly for assessing educational outcomes) so in some settings it would be important to delay this evaluation until after 6 years of age.

The benefit of being able to add independent teacher’s assessments and to more reliably access neurocognitive and behavioral outcomes is important for studies that are focused on IMPs that will affect brain development. Later school age outcomes should not necessarily be a part of the licensing process when no safety differences are detected at 2 years CGA. Individual sponsors and regulators should discuss whether school age outcomes are mandatory on an individual application basis or as a normal part of post-marketing analyses.

Figure 4 includes suggestions about the assessments that could be used when the investigational product has a known neurological target or when follow-up is conducted as part of a safety assessment. Evaluation at school age should comprise the domains assessed at 2 years, but with additional investigations:

- a formal test of general cognitive function; general cognitive scores (IQ) are closely related to gestational age at birth^25^
- more subtle aspects of cognitive function such as attention, executive function, processing speed and working memory^26^ using direct measures or parent report, e.g. the Behavior Rating Inventory of Executive Function (BRIEF).
- a measure of adaptive functioning to ensure that skills for daily living are included in standardized assessments, such as the Vineland Adaptive Behavior Scales (Vineland)
- behavior, social and emotional outcomes– using psychometric instruments that access behavior and emotional problems, inattention, autistic symptoms, and broader behavioral and psychiatric assessments^27^
- educational attainment – utilizing national attainment test results, teacher’s assessments of progress in the classroom, or standardized achievement tests (e.g. Wechsler Individual Achievement Tests (WIAT-III), Woodcock-Johnson IV Tests of Achievement). Special needs in the classroom are closely related to gestational age at birth^28^
- visual assessment including refraction and examination for strabismus
- other formal testing (e.g. lung function) as determined by the target of the investigational product
- in some countries (e.g. USA and Australia), school readiness assessments are performed which may be used to inform outcomes, but these are not in widespread use in other settings

All attempts to minimize dropout and encourage retention in the study should be taken, including regular contacts from the clinical team, birthday cards, newsletters, etc.

**References for Supplemental Material**

1. Edwards AD, Redshaw ME, Kennea N, et al. Effect of MRI on preterm infants and their families: a randomised trial with nested diagnostic and economic evaluation. *Archives of Disease In Childhood Fetal and Neonatal Edition* 2018;103(1):F15-F21.

2. Pierson CR, Al Sufiani F. Preterm birth and cerebellar neuropathology. *Seminars in Fetal & Neonatal Medicine* 2016;21(5):305-11.

3. Novak I, Morgan C, Adde L, et al. Early, Accurate Diagnosis and Early Intervention in Cerebral Palsy: Advances in Diagnosis and Treatment. *JAMA Pediatrics* 2017;171(9):897-907.

4. Palisano RJ, Hanna SE, Rosenbaum PL, et al. Validation of a model of gross motor function for children with cerebral palsy. *Physical Therapy* 2000;80(10):974-85.

5. Eliasson AC, Krumlinde-Sundholm L, Rosblad B, et al. The Manual Ability Classification System (MACS) for children with cerebral palsy: scale development and evidence of validity and reliability. *Developmental Medicine and Child Neurology* 2006;48(7):549-54.

6. Bayley N. Bayley-III Technical Manual. San Antonio, TX: PsychCorp 2006.

7. Ambalavanan N, Nelson KG, Alexander G, et al. Prediction of neurologic morbidity in extremely low birth weight infants. *Journal of Perinatology* 2000;20(8 Pt 1):496-503.

8. Green E, Stroud L, Bloomfield S, et al. Griffiths III: Griffiths Scales of Child Development (3rd Edition). Oxford: Hogrefe Ltd, 2015.

9. Linsell L, Johnson S, Wolke D, et al. Cognitive trajectories from infancy to early adulthood following birth before 26 weeks of gestation: a prospective, population-based cohort study. *Archives of Disease In Childhood* 2018;103(4):363-70.

10. Breeman LD, Jaekel J, Baumann N, et al. Attention problems in very preterm children from childhood to adulthood: the Bavarian Longitudinal Study. *Journal of Child Psychology and Psychiatry, and Allied Disciplines* 2016;57(2):132-40.

11. Achenbach TM, Rescoria LA. Manual for the ASEBA Preschool Forms and Profiles. Burlington VT: University of Vermont Department of Psychiatry, 2000.

12. Achenbach TM, Rescoria LA. Manual for the ASEBA School-Age Forms & Profiles. Burlington, VT: University of Vermont, Research Center for Children, Youth, & Families, 2001.

13. Carter AS, Briggs-Gowan MJ, Jones S.M., et al. The Infant-Toddler Social and Emotional Assessment (ITSEA): factor structure, reliability, and validity. *J Abnorm Child Psychol* 2003;31:495-514.

14. Karabekiroglu K, Briggs-Gowan MJ, Carter AS, et al. The clinical validity and reliability of the Brief Infant-Toddler Social and Emotional Assessment (BITSEA). *Infant Behav Dev* 2010;33:503-09.

15. Goodman R. The Strengths and Difficulties Questionnaire: a research note. *Journal of Child Psychology and Psychiatry, and Allied Disciplines* 1997;38(5):581-6.

16. Kuban KC, O'Shea TM, Allred EN, et al. Positive screening on the Modified Checklist for Autism in Toddlers (M-CHAT) in extremely low gestational age newborns. *The Journal of Pediatrics* 2009;154(4):535-40 e1. doi: 10.1016/j.jpeds.2008.10.011

17. Limperopoulos C, Bassan H, Sullivan NR, et al. Positive screening for autism in ex-preterm infants: prevalence and risk factors. *Pediatrics* 2008;121(4):758-65. doi: 10.1542/peds.2007-2158

18. Moore T, Johnson S, Hennessy E, et al. Screening for autism in extremely preterm infants: problems in interpretation. *Developmental Medicine and Child Neurology* 2012;54(6):514-20. doi: 10.1111/j.1469-8749.2012.04265.x

19. Marlow N. Measuring neurodevelopmental outcome in neonatal trials: a continuing and increasing challenge. *Archives of Disease In Childhood Fetal and Neonatal Edition* 2013; 98(6):F554-F58

20. Report of a BAPM/RCPCH working group. Classification of Health Status at 2 years as a perinatal outcome. London: BAPM, 2008.

21. Johnson S, Moore T, Marlow N. Using the Bayley-III to assess neurodevelopmental delay: which cut-off should be used? *Pediatric Research* 2014;75(5):670-4. doi: 10.1038/pr.2014.10

22. Doyle LW, Crowther CA, Middleton P, et al. Magnesium sulphate for women at risk of preterm birth for neuroprotection of the fetus. *The Cochrane database of systematic reviews* 2009(1):CD004661.

23. Roberts G, Anderson PJ, Doyle LW, et al. The stability of the diagnosis of developmental disability between ages 2 and 8 in a geographic cohort of very preterm children born in 1997. *Archives of Disease In Childhood* 2010;95(10):786-90.

24. Breeman LD, Jaekel J, Baumann N, et al. Preterm Cognitive Function Into Adulthood. *Pediatrics* 2015;136(3):415-23.

25. Kerr-Wilson CO, Mackay DF, Smith GC, et al. Meta-analysis of the association between preterm delivery and intelligence. *Journal of Public Health* 2012;34(2):209-16.

26. Mulder H, Pitchford N, Hagger M, et al. Development of Executive Function and Attention in Preterm Children: A Systematic Review. *Developmental Neuropsychology* 2009;34(4):393-421.

27. Johnson S, Marlow N. Preterm Birth and Childhood Psychiatric Disorders. *Pediatric Research* 2011;69(5):11R-18R.

28. MacKay DF, Smith GC, Dobbie R, et al. Gestational age at delivery and special educational need: retrospective cohort study of 407,503 schoolchildren. *PLoS Medicine* 2010;7(6):e1000289.
